# Deep Brain Stimulation Target Selection in Co-Morbid Essential Tremor and Parkinson’s Disease

**DOI:** 10.5334/tohm.62

**Published:** 2020-07-08

**Authors:** Anant Wadhwa, Sara Schaefer, Jason Gerrard, Wissam Deeb, Michael S. Okun, Amar Patel

**Affiliations:** 1 Department of Neurology, Yale School of Medicine, Yale University, New Haven, CT, US; 2 Department of Neurosurgery, Yale School of Medicine, Yale University, New Haven, CT, US; 3 Fixel Center for Neurological Diseases, Program for Movement Disorders and Neurorestoration, Department of Neurology, University of Florida, Gainesville, FL, US

**Keywords:** Essential Tremor, Parkinson’s disease, Deep Brain Stimulation

## Abstract

**Clinical Vignette:**

A 64-year-old man with essential tremor (ET) and Parkinson’s disease (PD) presented with medically refractory, large amplitude, debilitating rest and action tremor in his extremities.

**Clinical Dilemma:**

Ventral intermediate nucleus of the thalamus (VIM) deep brain stimulation (DBS) improves tremor in ET and PD but does not ameliorate bradykinesia and rigidity in PD. The comparative efficacy of subthalamic nucleus (STN) DBS in managing action ET tremor remains unclear.

**Clinical Solution:**

Bilateral STN was selected as the DBS target. Moderate improvement in rest tremor and mild improvement in action tremor were noted following initial programming.

**Gap In Knowledge:**

There are no head-to-head trials to guide DBS target selection in patients with both ET and PD. Current evidence is limited to a few small head-to-head trials that have demonstrated equivalent efficacy in tremor reduction in PD patients using VIM as DBS target and in ET patients using STN.

**Expert Commentary:**

Due to limited evidence, DBS treatment of complex cases, such as combined Parkinson’s disease and essential tremor, remains based on expert consensus at each institution. Further multi-approach efforts, using imaging, electrophysiologic, and animal data, will be needed to answer the identified gap in knowledge.

**Highlights:**

There is limited evidence to guide deep brain target selection in patients with essential tremor and Parkinson’s disease. We review existing literature and propose strategies to manage tremor in these patients.

## Clinical Vignette

**Figure 1:**
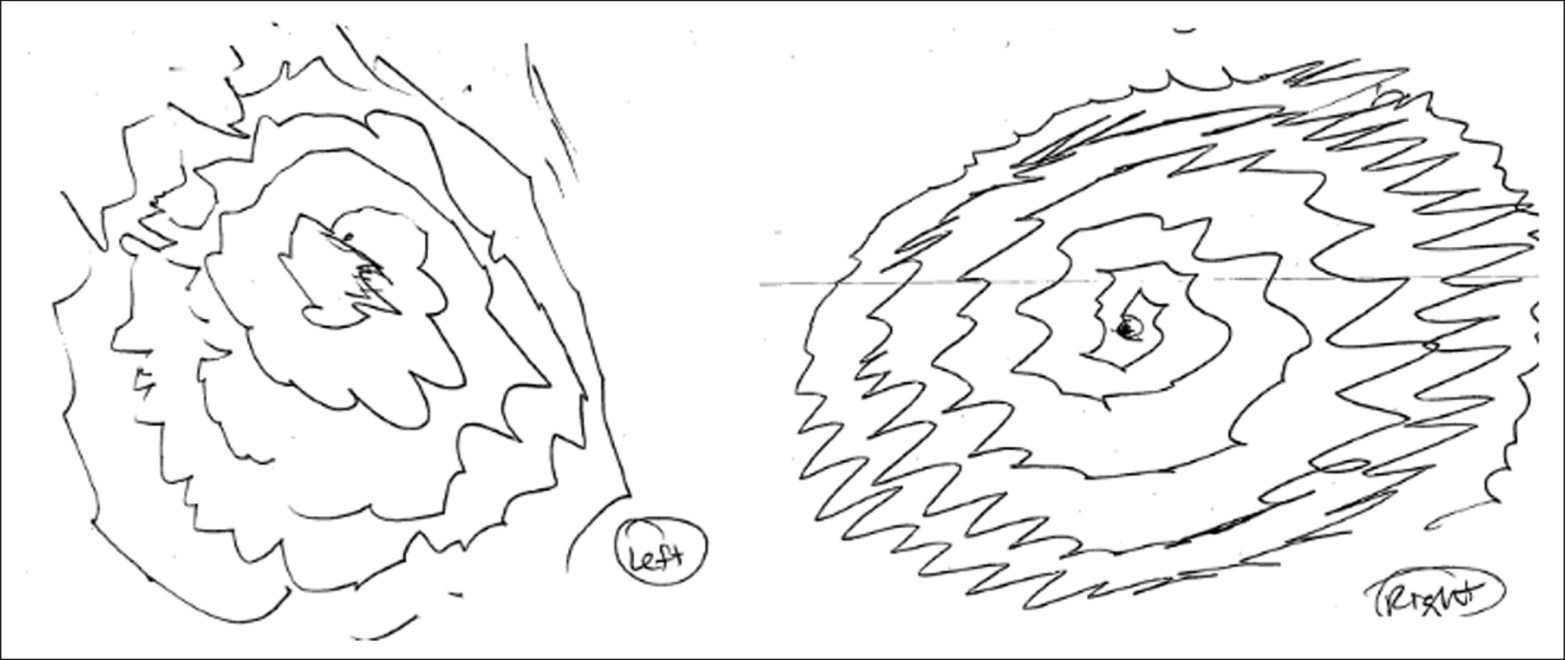
**Spirals.** Left and Right hand pre-operative spirals.

A 64-year-old right-handed male with essential tremor (ET) since the age of 18 presented for management of debilitating bilateral upper extremity action tremor. As a result, he had difficulty using his hands for eating, buttoning his clothes, and shaving. One year before presentation, he had also developed left lower extremity resting tremor. He had failed propranolol due to a lack of efficacy and could not tolerate primidone due to adverse effects. In addition to bilateral upper extremity action tremor (see Figure 1 for spiral drawing), examination showed rest tremor in both upper and lower extremities, chin tremor, hypomimia, hypometric saccadic eye movements, along with reduced arm swing and stride length when walking. Bradykinesia was present bilaterally but could not be accurately quantified due to severe overlying tremor. A clinical diagnosis of ET and Parkinson’s disease (PD) was made, based on the long-standing history of action tremor with onset in the upper extremities (ET), and more recent development of asymmetric rest tremor, bradykinesia and hypomimia (PD) [1]. A trial of a low dose of carbidopa-levodopa did not offer many benefits and was subsequently discontinued per patient preference. He was thus referred for deep brain stimulation (DBS) to address refractory tremor.

## Clinical Dilemma

DBS is recommended for PD patients with medically refractory tremor or motor fluctuations. Studies have shown comparable improvement in motor symptoms with stimulation of either the subthalamic nucleus (STN) or globus pallidus interna (GPi) [2]. Stimulation of the ventral intermediate nucleus of the thalamus (VIM) has demonstrated efficacy in the reduction of PD tremor as well [3,4,5,6]; however, thalamic stimulation does not meaningfully address bradykinesia and rigidity in PD [5,7]. Moreover, ET patients with medically refractory tremor are candidates for DBS, with VIM stimulation the traditionally favored target [8]. Stimulation of the STN has also demonstrated efficacy in the management of ET tremor in some patients, although only in limited cases series and reports [9,10,11]. While there is consensus on the stimulation targets for PD and ET, limited data is demonstrating a clear benefit of one target over others for patients with both disorders.

## Clinical Solution

The patient was discussed within the multidisciplinary DBS team, consisting of a movement disorders neurologist, functional neurosurgeon, and neuropsychologist. The patient was counseled regarding the risks and benefits of DBS and the choice of potential targets. Bilateral STN DBS using a conventional platform was offered. He underwent surgery using microelectrode recording (MER) guidance, without complication. Intraoperative recordings were robust and typical of STN. The patient demonstrated significant improvement in bradykinesia during intraoperative bipolar stimulation bilaterally, with milder improvement in tremor. Postoperative head CT revealed appropriate lead placement. After initial DBS programming, there was a moderate improvement in resting tremor and a slight improvement in action tremor, complicated by mild adverse effects of hypomania and dyskinesia (see Video 1).

**Video 1:** **Pre and post-operative exam.** Pre-operative assessment of tremor followed by post-operative tremor assessment during initial programming, complicated by hypomania and dyskinesia. Medtronic Activa PC Right STN settings: 1–, case+; 2.5 volts, 60 microseconds, 180 Hz. Left STN settings: 9–c+, 2.5 volts, 60 microseconds, 180 Hz. Lower stimulation voltages and frequencies were associated with poor tremor response. More dorsal active contact configurations were associated with reduced psychiatric adverse effects, though tremor benefit remained inadequate.

## Gaps in Knowledge

Direct evidence for appropriate target selection in patients with dual ET/PD is lacking. Clinicians can infer some guidance on target selection in these patients from evidence analyzing the effects of DBS on tremor in PD alone. A network meta-analysis by Mao et al., focusing on tremor-predominant PD subjects, noted similar changes in tremor scores between the STN, GPi and VIM in the medication “on” state [GPi –3.9 (95% CI –7.0 to –0.96); STN –3.1 (–5.9 to –0.38); VIM –1.9 (–17 to 13)]. However, in the medication “off” state, VIM DBS showed improved tremor control over both STN and GPi stimulation [GPi –8.5 (95% CI –19 to 1.7)); STN –9.1 (–18 to –0.13); Vim –17 (–33 to –2.6)] [12]. Cury et al. reported similar improvements in PD tremor in 54 patients with VIM DBS [6]. These benefits were sustained at a 10-year follow-up in a subgroup of 7 patients. DBS of the posterior subthalamic area (PSA) has also shown promise in treating PD tremor. Kitagawa et al. reported a 78.3% improvement in tremor in 8 PD patients two years after surgery [13]. Besides, rigidity improved by 92.7% and akinesia by 65.7%, with notable improvements in handwriting, posture, and gait as well.

These studies did not differentiate the relative type of tremor improvement (e.g., resting vs. action tremor). This information is vital in target selection, since rest tremor is often not functionally disabling, and its improvement would not necessarily reflect an improvement in a patient’s quality of life. Parihar et al. retrospectively reviewed PD patients with resting, postural, and action tremor who received STN (10 subjects) or VIM (8 subjects) DBS [14]. Resting and postural/action tremor improved 91% and 72%, respectively, in the VIM group versus 89% and 68%, respectively, in the STN group. With this limited retrospective evidence, STN, GPi, and VIM stimulation may have equivalent effects on both rest and action tremor improvement in PD.

Clinicians can also infer some guidance on target selection in ET/PD patients by analyzing the effects of “non-traditional” ET DBS targets on tremor reduction. Stimulation of the STN and caudal zona incerta (cZI) have both demonstrated tremor reduction in ET. In a head-to-head comparison of STN and VIM stimulation in ET patients, Lind et al. reported that 12 out of 21 patients demonstrated superior tremor control on STN stimulation during intraoperative testing when compared to VIM [10]. On long term follow up ranging from 1–9 years, these 12 patients who received STN DBS continued to demonstrate sound tremor reduction. In another study by Plaha et al., 4 ET patients showed significant improvement in postural/kinetic tremor (80.1% reduction in the Fahn-Tolosa-Marin Tremor Rating Scale) when examined a year after bilateral STN DBS surgery [8]. A review of 44 ET patients who received either VIM or cZI DBS demonstrated more significant tremor reduction with cZI stimulation (–2.2 ± 1.2 point reduction in the Washington Heights-Inwood Genetic Study of Essential Tremor (WHIGET) scale in the cZI group as compared to –1.2 ± 1.4 in the VIM group) [15]. However, another study of 47 ET patients showed that although tremor reduction between the cZI and VIM was comparable in the first two years after surgery, the cZI stimulation benefit seemed to wane 3–4 years after surgery [16]. While these studies reveal the potential for using targets like STN and cZI for ET, there is a dearth of head-to-head comparisons with VIM stimulation and no randomized studies.

There are two broad scenarios in which a patient with ET/PD might need DBS – (i) The patient who had received DBS for ET and subsequently developed PD for which a new target is required; and (ii) the patient who has hitherto not received DBS for ET, but later developed PD and would benefit from DBS for both ET and PD symptom management.

In the patient who develops PD after receiving DBS for ET, it is essential to determine the motor response to medical management. If motor symptoms of PD improve with medications, additional surgical considerations may not be necessary. Conversely, the patient who does not respond to medical management (refractory tremor) or develops PD motor fluctuations would fit the first (i) clinical scenario. A few strategies have been reported to address refractory PD tremor treatment. Concurrent stimulation of VIM and STN has been published in a patient with ET/PD, with initial bilateral VIM DBS followed by rescue leads for bilateral STN DBS after the onset of parkinsonism three years after the initial surgery [17]. This was well tolerated and led to an improvement of tremor and other motor symptoms of PD. Interestingly, there was an additive effect on tremor scores with simultaneous stimulation of both VIM and STN. Concerns regarding this strategy would be subjecting the patient to a second surgery and the potential for worsening side effects with concomitant stimulation of four targets, the long-term effects of which have not been studied. STN stimulation may contribute to dysarthria, weight gain, and postural instability [18]; whereas, VIM stimulation side effects include paresthesia, tonic muscle contractions, and also dysarthria [19]. Unilateral VIM DBS, followed by contralateral STN lead placement, is another strategy that has been reported in a case of ET/PD [20]. The obvious flaw with this strategy is that it would be useful in only a select group of ET/PD patients for whom rigidity and bradykinesia remain unilateral.

The second (ii) clinical scenario outlined above includes the current case under discussion. Given the potential benefit demonstrated by STN DBS in the management of ET postural/kinetic tremor, it may be considered as a possible target for ET. It would be expected to address the motor manifestations of PD, were they to worsen subsequently. However, a larger head-to-head trial is needed to compare its efficacy to VIM stimulation for the management of tremor in patients with dual ET and tremor-predominant PD.

Future strategies may not be limited to single target stimulation. Newer lead technologies with longer ranges of active stimulation and independent current control may allow for simultaneous stimulation of the VIM and STN and/or cZI using a single electrode, as has been described by Falconer et al. [21]. Closed-loop DBS may offer adaptive stimulation options. Similarly, phase-controlled DBS, which has been piloted in thalamic DBS for tremor, may show superior options for tremor control in either ET or PD [22].

## Expert Commentary

DBS is an established treatment option for multiple movement disorders such as Parkinson’s disease, essential tremor, and dystonia. However, as this case exemplifies, the evidence guiding the management of more complex clinical presentations, with either mixed etiologies or complex clinical signs and symptoms, remains scant [23]. Due to limited evidence, DBS treatment of complex cases, such as combined Parkinson’s disease and essential tremor, remains based on expert consensus at each institution. Different medical centers tend to use different approaches such as simultaneous or staged multi-target DBS, asymmetric targeting, as well as lead positioning to straddle a couple of targets. The decision relies mainly on the age of the patient, DBS lead trajectory, clinical manifestations, impairment, individualized goals, and projected evolution of symptoms. For instance, a patient with a combined ET and PD diagnosis whose rigidity and bradykinesia are medically well-controlled but with refractory and impairing ET and/or PD tremor would benefit from Vim DBS. It is important to counsel the patient that a rescue STN DBS lead might be needed in the future if motor fluctuations or dyskinesias develop.

This highlights an overarching gap in knowledge – the limited understanding of the dedicated circuits and the physiologic effects of DBS. In addition to cZI and PSA, the centromedian parafascicular nucleus has been used as a rescue in tremor-resistant STN-DBS cases based on the possible role of this nucleus in the tremor circuits [24]. Ongoing electrophysiologic, functional, and tractographic evaluations continue to shed some light on the physiologic differences and commonalities among these co-occurring disorders and will improve our understanding and choice of targets [25]. Furthermore, technological advancement will allow individualized maps, thus improving decision making [26]. Finally, the use of wearable sensors can help improve the characterization of the tremors and other movement signs and the effect of the different DBS targets [27].

Further multi-approach efforts, using imaging, electrophysiologic, and animal data, will be needed to answer the identified gap in knowledge.
